# Trigeminal Medullary Dorsal Horn Neurons Activated by Nasal Stimulation Coexpress AMPA, NMDA, and NK1 Receptors

**DOI:** 10.1155/2013/152567

**Published:** 2013-12-08

**Authors:** P. F. McCulloch, K. M. DiNovo, D. J. Westerhaus, T. A. Vizinas, J. F. Peevey, M. A. Lach, P. Czarnocki

**Affiliations:** Department of Physiology, Chicago College of Osteopathic Medicine, Midwestern University, 555 31st Street, Downers Grove, IL 60515, USA

## Abstract

Afferent information initiating the cardiorespiratory responses during nasal stimulation projects from the nasal passages to neurons within the trigeminal medullary dorsal horn (MDH) via the anterior ethmoidal nerve (AEN). Central AEN terminals are thought to release glutamate to activate the MDH neurons. This study was designed to determine which neurotransmitter receptors (AMPA, kainate, or NMDA glutamate receptor subtypes or the Substance P receptor NK1) are expressed by these activated MDH neurons. Fos was used as a neuronal marker of activated neurons, and immunohistochemistry combined with epifluorescent microscopy was used to determine which neurotransmitter receptor subunits were coexpressed by activated MDH neurons. Results indicate that, during nasal stimulation with ammonia vapors in urethane-anesthetized Sprague-Dawley rats, activated neurons within the superficial MDH coexpress the AMPA glutamate receptor subunits GluA1 (95.8%) and GluA2/3 (88.2%), the NMDA glutamate receptor subunits GluN1 (89.1%) and GluN2A (41.4%), and NK1 receptors (64.0%). It is therefore likely that during nasal stimulation the central terminals of the AEN release glutamate and substance P that then produces activation of these MDH neurons. The involvement of AMPA and NMDA receptors may mediate fast and slow neurotransmission, respectively, while NK1 receptor involvement may indicate activation of a nociceptive pathway.

## 1. Introduction

The diving response, an autonomic reflex characterized by apnea, bradycardia, and increased peripheral vascular resistance, is initiated through nasal stimulation or when animals submerge under water [1]. Afferent information involved in initiating this response projects from the nasal mucosa to the spinal trigeminal nucleus via the anterior ethmoidal nerve (AEN), a branch of the ophthalmic division of the trigeminal nerve [2]. The central projections of the AEN terminate within the spinal trigeminal nucleus caudalis [3]. This area is also known as the medullary dorsal horn (MDH) due to its functional and anatomical similarities with the spinal dorsal horn (SDH) [4]. Second-order neurons within the MDH are activated both during voluntary diving in conscious animals [5] and nasal stimulation in anesthetized animals [6–8]. It is likely that excitatory amino acids serve as neurotransmitters at synapses within the MDH that are part of this response, as injection of kynurenate, a general excitatory amino acid antagonist, into the MDH abolishes the cardiorespiratory responses to nasal stimulation [9]. More specifically, it is likely that glutamatergic neurotransmission in the MDH after nasal stimulation is at least partially dependent on the NMDA subtype of glutamate receptors [8]. However, other glutamate receptor subtypes and other neurotransmitters/neuromodulators may also be involved in the neurotransmission between the AEN and MDH secondary neurons.

Glutamate is the major excitatory neurotransmitter in the central nervous system [10–12]. It activates both ligand-gated ionotropic glutamate receptors and G-protein-coupled metabotropic glutamate receptors [10, 12]. Based on their agonist specificities, ionotropic receptors, those receptors that are directly coupled to cation channels, are divided into *α*-amino-3-hydroxy-5-methyl-4-isoxazole-propionate (AMPA), kainate, and N-methyl-D-aspartate (NMDA) receptors [10, 12, 13]. Ionotropic glutamate receptors are formed from heteromeric subunit assemblies that have different physiological and pharmacological properties [12]. The subunit composition determines the biophysical properties of the receptor, and the subunits that comprise these are specific for each of the three ionotropic glutamate receptor families [11, 13]. Ionotropic glutamate receptors are formed from tetrameric assemblies of different subunits [12], and the four subunits are arranged with three transmembrane-spanning and one pore-lining domain [12, 14].

AMPA and kainate receptors collectively are known as non-NMDA receptors [10, 13]. AMPA receptors mediate fast excitatory neurotransmission in most synapses within the CNS [10, 12, 14] and are typically concentrated at the postsynaptic sites of excitatory synapses [15]. Subunits that compose AMPA receptors include GluA1, GluA2, GluA3, and GluA4 [10, 12, 14]. Kainate receptors exhibit fast activation similar to AMPA receptors but are thought to fulfill more of a neuromodulatory role in the CNS [16]. Five kainate receptor subunits have been identified: GluK1, GluK2, GluK3, GluK4, and GluK5 [10, 14]. Kainate and AMPA receptors subunits can coexist in the same neurons but do not appear to coassemble with each other [10]. At most central synapses, both AMPA and NMDA receptors are activated during synaptic transmission. AMPA receptors mediate fast neurotransmission while neurotransmission mediated by NMDA receptors occurs more slowly and lasts much longer [10, 13]. The fundamental NMDA receptor subunit is GluN1, with modulatory subunits being GluN2A, GluN2B, GluN2C, GluN2D, and GluN3 [10, 12, 14].

Neuropeptides and kinins are important messengers in the nervous system [17]. The neurokinin substance P (SP), an 11-amino acid peptide [18–20], was identified as the first member of the tachykinin family [19–21]. The neurokinin receptor NK1 is preferentially activated by SP and is a G-protein-coupled receptor with seven transmembrane-spanning domains [18, 22]. SP, acting at NK1 receptors, elicits excitatory effects as a neurotransmitter or neuromodulator in both the central and peripheral nervous systems [17, 18], although the functional role of tachykinins could be related to an interaction with glutamate acting at NMDA receptors at the postsynaptic site [23, 24]. SP and NK1 receptors function in slow nociceptive neurotransmission, primarily in tissue injury and inflammation rather than in acute pain [17, 21, 24].

The present study was designed to characterize the MDH neurons involved in mediating the cardiorespiratory changes induced after nasal stimulation by determining the types of neurotransmitter receptors that are expressed by these activated MDH neurons. Fos was used as a neuronal marker of activated neurons [25], and immunohistochemistry was used to determine which neurotransmitter receptor subunits were coexpressed by activated MDH neurons. The identification of which type of ionotropic glutamate receptor (AMPA, kainite, or NMDA) and whether SP is involved would help describe the specifics of the afferent signal and the possible integration of that signal within second-order MDH neurons.

## 2. Materials and Methods

All experimental procedures were approved by the Midwestern University IACUC. Male Sprague-Dawley rats (258–563 g; *N* = 37) were obtained from a commercial vendor (Harlan). Brain tissue from some animals was used to test more than one Fos/neurotransmitter receptor combination (see below).

### 2.1. Stimulation of Nasal Passages

The nasal passages of rats were stimulated with ammonia vapors to activate MDH neurons. To reduce animal usage, control experiments using unstimulated animals were not repeated here, as unstimulated animals show neither cardiorespiratory changes nor activation of MDH neurons [6, 7]. For complete experimental details, see Rybka and McCulloch [6]. Briefly, rats were initially anesthetized with 5% isoflurane (in 95% O_2_/5% CO_2_) and then transferred to a nose cone delivering 2-3% isoflurane. The right femoral artery and vein were cannulated to record blood pressure and administer drugs, respectively. The trachea was cannulated caudally to enable ventilation, and rostrally to aid in stimulation of the nasal passages. Respiratory rate was monitored through thermal sensing of air flow in the caudal tracheal cannula. After surgery was complete, isoflurane was withdrawn as urethane (1300 mg/kg, iv) was slowly injected. Rats rested for approximately 1 hr to achieve a stable plain of urethane anesthesia before the start of experiments. Body temperature was maintained at 37 ± 1°C. Electronic signals for respiration and arterial blood pressure (BPa) were recorded, stored and analyzed using appropriate computer software (Spike2, CED). Heart rate (HR) was determined from pulse pressure intervals.

A stimulation trial consisted of placing a cotton swab soaked in ammonia 2-3 mm in front of the external nares for 5 s. A suction pump connected to the rostral tracheal cannula gently drew ammonia vapors through the nasal passages. Stimulations occurred every 5 min for 2 hr for a total of 24 trials. A 1 hr wait followed the final stimulation to allow activated neurons to produce Fos. At the end of experiments rats were euthanized with concentrated sodium pentobarbital (0.3 mL Sleepaway, iv; Fort Dodge), followed by a transcardiac perfusion with a 300 mL phosphate buffered saline (PBS) solution containing 0.25% procaine and then 300 mL 4% paraformaldehyde. Finally, the brains were removed and stored in a PBS solution containing 4% paraformaldehyde and 20% sucrose. The brains postfixed for a minimum of 24 hr at 4°C, and the brainstems were then cut transversely at 40 or 50 *μ*m using a freezing microtome.

### 2.2. Immunohistochemistry

PBS was used for all immunohistochemical washes. Free floating (1 : 3 series) brainstem sections were blocked in 10% normal donkey serum for 1 hr. The sections were incubated overnight with an anti-Fos primary antibody (see Table 1). The next day the sections were blocked for 1 hour in 10% bovine serum albumin (BSA). Next, the sections were incubated with a fluorescent-tagged secondary antibody directed against the Fos primary antibody for 2 hr. The sections were then blocked with 10% normal donkey serum for 1 hour. The sections were then incubated overnight in a second primary antibody directed against a specific neurotransmitter receptor or receptor subunit (see Table 1). The next day the sections were again blocked for 1 hr in 10% BSA. Next, the sections were incubated with a fluorescent-tagged secondary antibody directed against the primary receptor antibody for 2 hr. Lastly, sections free floating in PBS were organized into serial order, mounted on clean slides, and coverslipped with a buffered glycerol mounting solution. All tissue processing occurred in minimal lighting in order to minimize fluorescent fading.

On some occasions tissue sections were incubated overnight with a cocktail of primary antibodies for both Fos and the specific neurotransmitter receptor (see Table 1). The following day the sections were blocked for 1 hr in 10% BSA. Next, the sections were incubated with a fluorescent-tagged secondary antibody directed against the Fos primary antibody for 2 hr. The sections were then blocked for 1 hr in 10% BSA. Next, the sections were incubated with a fluorescent-tagged secondary antibody directed against the receptor primary antibody for 2 hr. The sections were then organized into serial order, mounted, and coverslipped.

### 2.3. Microscopy

**Figure 1:**
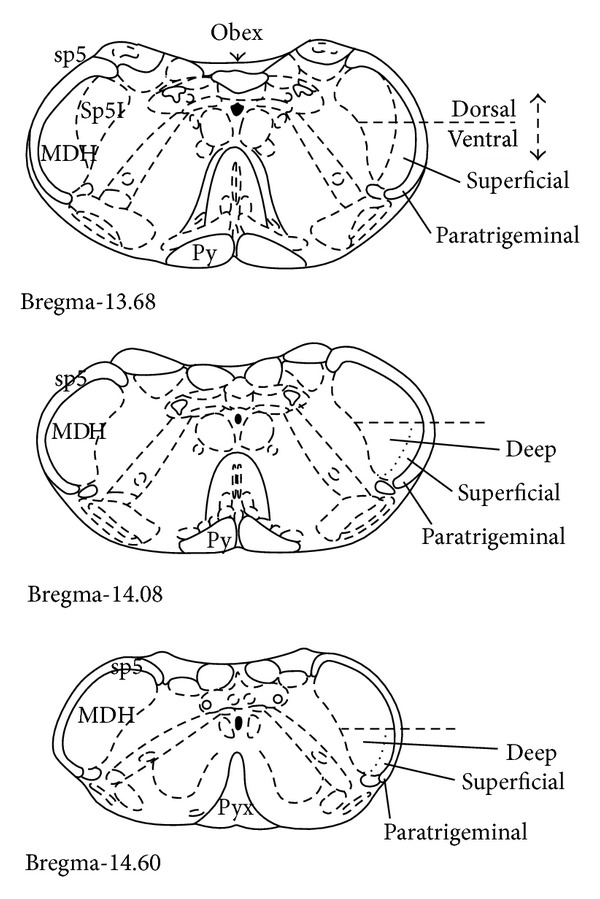
After stimulation of the nasal passages with ammonia vapors, Fos-positive cells were counted bilaterally in the ventral portion of the medullary dorsal horn, between the pyramidal decussation (Bregma-14.60) and obex (Bregma-13.68). Superficial is MDH laminae I&II; Deep is MDH laminae III–V; Paratrigeminal neurons are located within spinal trigeminal tract. Cross-sections from Paxinos and Watson [26]. Abbreviations: MDH, medullary dorsal horn; Py, pyramidal tract; Pyx, pyramidal decussation; sp5, spinal trigeminal tract, Sp5I, spinal trigeminal nucleus interpolaris.

A Nikon Eclipse E600 microscope with epifluorescent attachment was used to visualize the right and left MDH (Figure 1). An average of 20.13 ± 1.01 MDH hemisections (left: 10.11 ± 0.58; right: 9.98 ± 0.51) was used to visualize each of the 61 primary and secondary antibody pairings (see Table 1). Tissue was first inspected for the presence of Fos in the nucleus of the neuron (indicating activation of that neuron) and then for the neurotransmitter receptor or receptor subunit on the cellular membrane (indicating expression of that neurotransmitter receptor by that neuron). The number of single-labeled (Fos only) and colocalized (Fos-positive and neurotransmitter receptor positive) neurons were counted bilaterally within the ventral MDH (both superficial (laminae I and II) and deep regions (laminae III–V)). Neurons within the ventral paratrigeminal nucleus, located adjacent to the MDH within the spinal trigeminal tract, were also counted. Fos-positive neurons in other brain regions were not counted. To aid in the counting and to reduce fluorescent fading of the tissue, 10x photomicrographs were taken through red and green cube filters with a digital camera (Q-Imaging) and associated imaging software (Northern Eclipse). Subsequent microscopic analysis at 20 or 40x and inspection of photomicrograph overlays of the red and green images verified the presence of single-labeled or colocalized neurons.

### 2.4. Data Presentation

HR (bpm; beats/min), BPa (mm Hg), and respiratory rate (breaths/min) are presented as mean ± standard error (SE). Cardiovascular data were analyzed by comparing pretrial, trial, and posttrial values. Photomicrographs were adjusted using ImageJ (v1.43 g, NIH), and figures were composed and labeled using CorelDraw (Corel). Neuronal data are presented as number of Fos-positive neurons ± SE per hemisection and as the percentage of Fos-positive neurons that coexpressed the specific neurotransmitter receptor or receptor subunit. Statistical differences were tested with Repeated Measures One-Way ANOVAs (SigmaStat, SPSS), with *P* < 0.05 set as the level of significance.

## 3. Results

**Figure 2:**
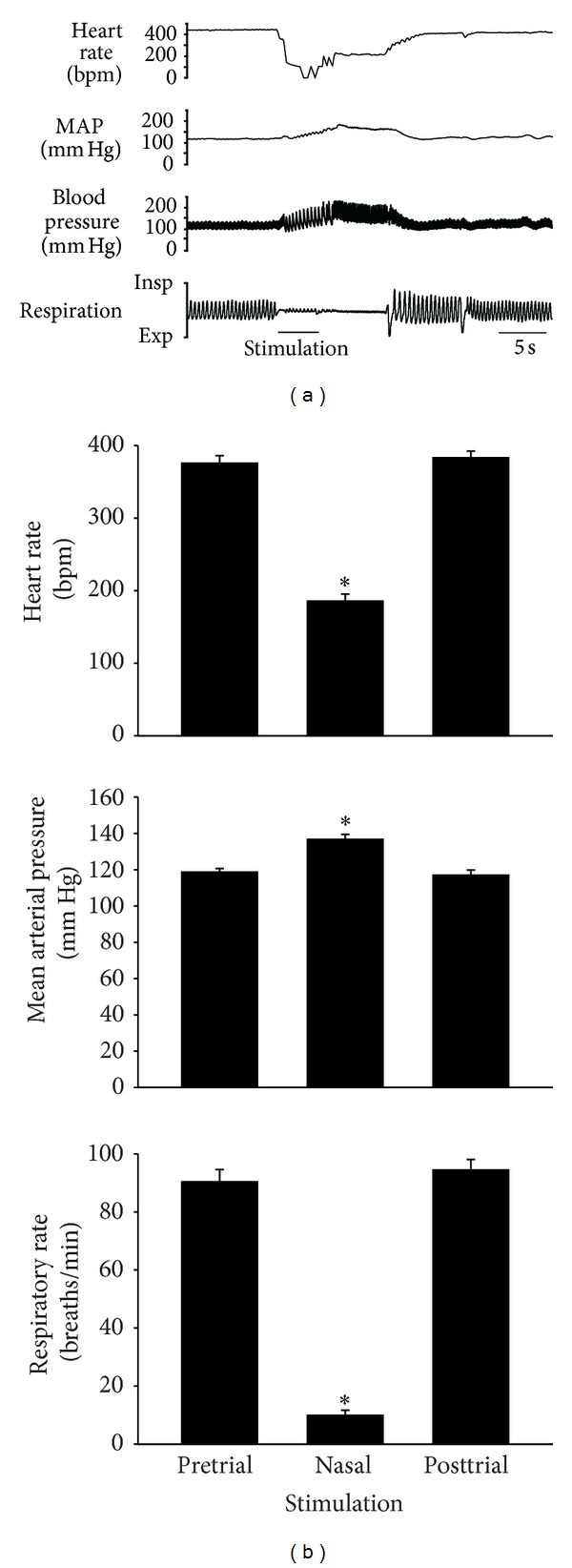
(a) Original traces showing cardiorespiratory responses to stimulation of the nasal passages with ammonia vapors (bar under trace). MAP is mean arterial pressure. (b) Heart rate, mean arterial blood pressure, and respiratory rate (mean ± SE) before, during, and after nasal stimulation. *Significantly different from both pretrial and posttrial.

Stimulation of the nasal passages with ammonia vapors produced an immediate and intense cardiorespiratory response that included bradycardia, an increase in arterial blood pressure, and apnea (Figure 2(a)). During the 5 s of nasal stimulation, HR decreased significantly from 375 ± 11 to 185 ± 10 bpm and mean BPa significantly increased from 119 ± 2 to 137 ± 3 mm Hg (Figure 2(b)). Apnea lasted an average of 10.1 ± 0.6 s, but if apnea was not present, respiratory rate significantly decreased from 90 ± 4 to 10 ± 2 breaths/min (Figure 2(b)).

The total Fos labeling (Fos only neurons plus Fos-positive neurons colocalized with a neurotransmitter receptor) was significantly greater in the superficial MDH, compared with the total Fos labeling in the deep MDH and paratrigeminal nucleus. Within each MDH hemisection, there were 11.74 ± 1.07 total Fos-positive neurons in the superficial MDH, compared with 3.61 ± 0.42 and 1.43 ± 0.18 Fos-positive neurons within the deep MDH and paratrigeminal nucleus, respectively. Per hemisection, 69.9% of the activated neurons were located in the superficial MDH, with 21.5% and 8.5% located in the deep MDH and paratrigeminal nucleus, respectively.

**Figure 3:**
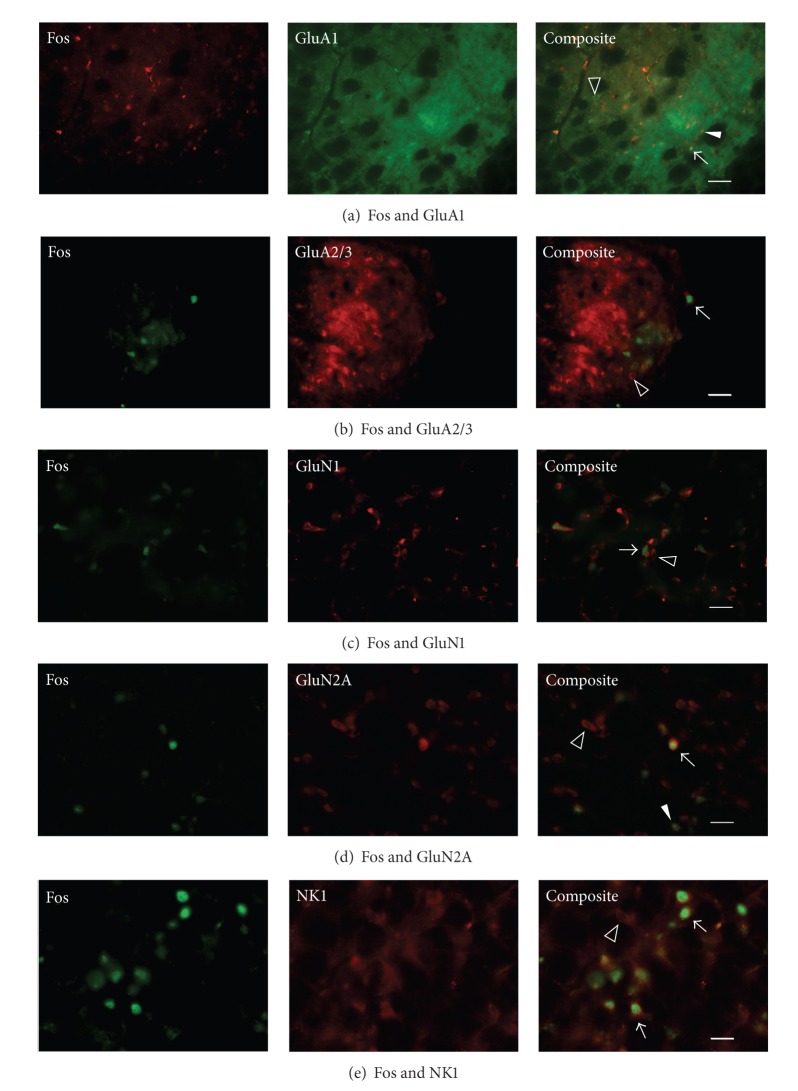
Fluorescent photomicrographic images showing individual Fos (left column), neurotransmitter subunit or receptor (middle column), and composite colocalized labeling (right column). Within the superficial MDH filled triangles indicate Fos-positive neurons, open arrowheads indicate Fos-negative but subunit or receptor positive neurons, and arrows indicate Fos-positive colocalized neurons. Scale bars: (a) 50 *μ*m, (b)–(e) 25 *μ*m.

The number of Fos only and colocalized Fos-positive neurons (and therefore the percent of colocalized neurons) depended upon which neurotransmitter receptor was immunolabeled (Table 2). Within the superficial MDH, Fos-positive neurons predominantly coexpressed AMPA glutamate receptors subunits GluA1 (95.8%) and GluA2/3 (88.2%), NMDA glutamate receptor subunits GluN1 (89.9%) and GluN2A (41.4%), and SP NK1 (64.1%) receptors (Figure 3, Table 2). Other AMPA, NMDA, and kainate receptor subunits were not coexpressed to any great extent by Fos-positive neurons (Table 2). Within the deep MDH, Fos-positive neurons predominantly coexpressed GluA1 (91.7%) and GluN1 (94.4%) subunits and to a lesser extent GluA2/3 (25.0%) and GluN2A (40.1%) subunits and SP NK1 (40.8%) receptors (Table 2). Within the paratrigeminal nucleus, Fos-positive neurons predominantly coexpressed GluA1 (93.1%), GluA2/3 (75.0%), GluN1 (95.5%), and GluN2A (57.3%) subunits and to a lesser extent SP NK1 (19.5%) receptors (Table 2).

## 4. Discussion

Stimulation of the nasal mucosa of rats with ammonia vapors produces prolonged apnea, a significant decrease in HR, and a significant increase in mean arterial pressure. As part of the central neuronal circuitry of this response, there is activation of neurons within the spinal trigeminal nucleus, specifically the MDH. The novel results from the present study indicate that during stimulation of the nasal mucosa of rats with ammonia vapors, many of these activated MDH neurons coexpress the AMPA glutamate receptor subunits GluA1 and GluA2/3 and the NMDA glutamate receptor subunits GluN1 and GluN2A, and a significant proportion coexpresses NK1, the receptor for SP.

The cardiorespiratory responses resulting from repetitive stimulation of the nasal mucosa with ammonia vapors has been described previously in both rats [3, 6] and muskrats [7]. Consistent with these results, we found that nasal stimulation produced apnea, significant bradycardia, and a significant increase in BPa. The efferent aspects of this reflex response may be mediated by brainstem autonomic areas, including the nucleus tractus solitarius, the ventrolateral medulla, the A5 area, and the peribrachial complex [27]. The AEN innervates the nasal mucosa and is essential for the initiation of the afferent portion of this nasopharyngeal response [6]. The AEN projects primarily to the ipsilateral superficial portions of the ventral MDH and the paratrigeminal nuclei located within the ventral spinal trigeminal tract [3, 28]. Neurons in these regions express Fos after either nasal stimulation [6, 8, 29] or voluntary diving [5], and a similar pattern of Fos labeling was found within the MDH and paratrigeminal nucleus after nasal stimulation in the present study. Central projections of the AEN colocalize with MDH neurons activated by nasal stimulation, and the density of AEN terminal projections positively correlates with the rostral-caudal location of activated MDH neurons [3]. Presumably the afferent signals carried by the AEN activate these secondary neurons within the MDH [3]. These MDH neurons then likely project to other brainstem locations that are important in the production of the cardiorespiratory responses to nasal stimulation [28].

*c-fos* is an immediate early gene that encodes transcription factors which can participate in long-term alteration of cellular function [30]. As such, the immunological detection of Fos, the protein product of *c-fos*, has been used as a marker for neuronal activation within the CNS [30]. This technique appears to be particularly useful in identifying the afferent limb of a reflex circuit such as the diving response [7]. However the temporal pattern of Fos production and decay is dependent upon the brain region being investigated and type of stimulation used [31]. The production of Fos within secondary MDH neurons that results from intermittent and repetitive stimulation of the nasal mucosa has been previously described [3, 5–8]. This protocol (2 hrs of stimulation trials plus a 1 hr wait) was chosen because Fos is produced within MDH neurons of animals receiving nasal stimulation but not within the MDH of unstimulated control animals [6, 7]. For consistency and to extend the findings from these previous results, the present experiment used an identical stimulation protocol to ensure Fos production within MDH neurons. However, Fos can be expressed with minutes of neuronal activation [32] and receptor trafficking to the cell surface [14, 22] may have been altered by the nasal stimulation itself. It is therefore possible that between activation of the cardiovascular response by nasal stimulation and the termination of the experiment after a 1 hr wait, there may have been an alteration in receptor distribution due to changes in receptor internalization or expression. Thus a potential limitation of our results may be that coexpression of Fos and receptors represents coexpression at the time of euthanasia rather than coexpression at the time of actual nasal stimulation. While this may be a possibility, we feel that the receptor coexpression reported in these experiments is representative of what occurs during nasal stimulation and therefore helps to characterize the MDH neurons involved in mediating the cardiorespiratory changes induced after nasal stimulation.

Given the caveat just stated above, the results from the present experiments indicate that secondary neurons within the ventral portion of the MDH that are activated by nasal stimulation with ammonia vapors express AMPA, NMDA and NK1 receptors, but not kainate receptors (Figure 3, Table 2). Secondary MDH neurons have previously been found to express both AMPA, and NMDA glutamate receptors [33, 34]. Because the AEN innervates the nasal passages, sends central projections to the MDH location, and induces activation of MDH neurons [3], and since these MDH neurons express AMPA, NMDA, and NK1 receptors (present study), our results suggest that the AEN releases glutamate as a neurotransmitter and SP as a cotransmitter/neuromodulator after stimulation of the nasal passages. Presumably glutamate and/or SP released by the AEN activate these secondary MDH neurons and induce them to produce Fos through activation of multiple glutamatergic and SP receptors.

Within the superficial layers (laminae I and II) of the MDH quantitative autoradiography indicates a high density of AMPA receptors [35]. With regards to AMPA receptor subunits, GluA1 is expressed moderately, GluA2/3 is intensely expressed, and GluA4 is not expressed [36]. In the present study, over 90% of MDH neurons activated by nasal stimulation (both within the superficial and deep MDH laminae, as well as in the adjacent paratrigeminal region) coexpress the glutamate receptor subunit GluA1 (Figure 3, Table 2). Additionally, GluA2/3 glutamate receptor subunits are coexpressed by over 75% of neurons within the superficial MDH and paratrigeminal region. Since GluA1, GluA2, and GluA3 are all subunits that form AMPA receptors, this suggests that AMPA receptors are present on these secondary MDH neurons. The involvement of AMPA receptors in the physiological responses to nasal stimulation has been suggested previously, as infusion of DNQX, an antagonist of non-NMDA glutamate receptors, into the spinal trigeminal nucleus reversibly eliminates the cardiorespiratory responses to nasal stimulation [37]. Since AMPA receptors mediate fast synaptic neurotransmission [10, 12, 14], this glutamatergic mechanism may mediate the fast cardiorespiratory reflex responses that result from nasal stimulation (Figure 2). Electrophysiological evidence indicates that neurons within lamina I of the MDH produce AMPA receptor-mediated excitatory postsynaptic currents after electrical stimulation of the spinal trigeminal tract [38].

There may also be a slower neurosynaptic mechanism within the MDH involving glutamate release by the central terminations of the AEN, as activated MDH neurons coexpress the NMDA receptor subunits GluN1 and GluN2A (Table 2). GluN1 receptor subunits are strongly expressed in the MDH [39, 40]. Previous results have indicated that NMDA receptors may be involved in producing the cardiorespiratory responses elicited by nasal stimulation. Kynurenic acid, an antagonist of the glycine binding site located on the GluN1 subunit [13, 41], abolishes the cardiorespiratory responses to nasal stimulation when infused into the MDH [9]. AP7, an NMDA receptor antagonist, reversibly eliminates the cardiorespiratory responses to nasal stimulation when injected into the spinal trigeminal nucleus [37]. Also, stimulation of the nasal mucosa with saline produces a significant increase in Fos-like immunoreactivity within the MDH, and 53% of these Fos-positive neurons are also immunoreactive for the GluN1 receptor subunit [8]. Finally, electrical stimulation of the spinal trigeminal tract produces NMDA receptor-mediated excitatory postsynaptic currents within lamina I MDH neurons [38]. These studies and the present results all suggest that glutamatergic neurotransmission in the MDH after nasal stimulation is at least partially dependent on NMDA receptors.

NK1 receptors were coexpressed by approximately 50% of activated MDH neurons within the superficial and deep MDH and by 20% of activated MDH neurons within the paratrigeminal nucleus. However it is possible that NK1 receptor internalization after activation by SP [19, 22] may have caused an underrepresentation of the number of activated neurons that coexpressed NK1 receptors. Within the dorsal horn glutamate and SP have been shown to coexist in primary afferent C-fibers [42] and are coreleased by primary afferent neurons involved in mediating nociception [23, 43]. NMDA receptors are frequently located in the postsynaptic targets of SP terminals and may play a role in the modulation of SP containing neurons [44]. Additionally, glutamate activated conductance in rat spinal dorsal horn neurons is enhanced by SP [23]. Many lines of evidence indicate that SP and NK1 receptors play an important role in nociception [17–20, 24], and pain associated with peripheral tissue or nerve injury involves NMDA receptor activation [45]. Because there is activation of MDH neurons expressing both NMDA and NK1 receptors, the present results suggest that at least part of the signal originating from the nasal passages that initiates the observed cardiorespiratory responses is nociceptive in nature. In support of this contention, approximately 65% of the muskrats AEN is composed of unmyelinated C-fibers, and 72% of the AEN myelinated fibers are of the A-*δ* small diameter type [46]. Because nociceptive pathways utilize small diameter fibers [47], the fact that the AEN contains roughly 90% small diameter fibers [46] suggests that the AEN may be involved in nociceptive signaling. Additionally, the superficial region of the dorsal horn receives direct input from myelinated and unmyelinated nociceptors [47] and was where 69.9% of the activated (Fos-positive) MDH neurons were located in the present study.

## 5. Conclusion

The present study has identified neurotransmitter receptors present on MDH neurons that are activated during nasal stimulation. Glutamate receptors include both AMPA and NMDA, but not kainate, subtypes. Additionally, many of the activated MDH neurons also coexpressed NK1 receptors. It is therefore likely that the terminal fibers of the anterior ethmoidal nerve that synapse with MDH neurons release glutamate and SP to activate these MDH neurons during nasal stimulation. The involvement of AMPA and NMDA receptors may mediate fast and slow neurotransmission, respectively, while NK1 receptors may indicate activation of a nociceptive pathway.

## 6. Highlights

1. Nasal stimulation produces apnea, bradycardia, and increased arterial blood pressure.
2. Neurons within trigeminal medullary dorsal horn are activated by nasal stimulation.
3. Activated trigeminal medullary dorsal horn neurons express AMPA, NMDA, and NK1 receptors.

## Figures and Tables

**Table 1:** Primary and secondary antibody combinations. Based on availability of primary and secondary antibodies and results obtained in preliminary experiments, antibodies, fluorescent color combinations, and dilutions were changed as necessary. For catalog product numbers, C: Chemicon; I: Invitrogen; M: Millipore; MP: Molecular Probes; and SC: Santa Cruz. For fluorescent tagged secondary antibodies, AF: AlexaFluor.

AMPA		
GluA1 (GluR1) and Fos (N = 6)		
	Fos (red)	GluA1 receptor subunit (green)
Primary Ab:	goat (SC, sc-52G); 1 : 1000	rabbit (M, AB1504); 1 : 10,000
Secondary Ab:	donkey (MP, A11058; AF594); 1 : 500	donkey (MP, A21206; AF488); 1 : 500
GluA2/3 (GluR2/3) and Fos (N = 4)		
	Fos (green)	GluA2/3 receptor subunits (red)
Primary Ab:	goat (SC, sc-52G); 1 : 2500	rabbit (C, AB1506); 1 : 2500
Secondary Ab:	donkey (MP, A11055; AF488); 1 : 1000	donkey (MP, A21207; AF594); 1 : 1000
GluA (GluR4) and Fos (N = 3)		
	Fos (red)	GluA4 receptor subunit (green)
Primary Ab:	goat (SC, sc-52G); 1 : 1000	rabbit (M, AB1508); 1 : 5000
Secondary Ab:	donkey (MP, A11058; AF594); 1 : 500	donkey (MP, A21206; AF488); 1 : 500
Kainate		
GluK1/2/3 (GluR5/6/7) and Fos (N = 3)		
	Fos (green)	GluK1/2/3 receptor subunits (red)
Primary Ab:	rabbit (SC, sc-52); 1 : 1000	mouse (M, MAB379); 1 : 5,000
Secondary Ab:	donkey (MP, A21206; AF488); 1 : 500	donkey (MP, A21203; AF594); 1 : 500
GluK4 (KA1) and Fos (N = 6)		
	Fos (green)	GluK4 receptor subunit (red)
Primary Ab:	goat (SC, sc-52G); 1 : 2000	rabbit (SC, sc-25700); 1 : 100
Secondary Ab:	donkey (I, A11055; AF488); 1 : 500	donkey (I, A21207; AF594); 1 : 1000
GluK5 (KA2) and Fos (N = 5)		
	Fos (green)	GluK5 receptor subunit (red)
Primary Ab:	goat (SC, sc-52G); 1 : 2000	rabbit (SC, sc-25701); 1 : 200
Secondary Ab:	donkey (I, A11055; AF488); 1 : 1000	donkey (I, A21207; AF594); 1 : 1000
NMDA		
GluN1 (NR1, NMDA*ζ*1) and Fos (N = 5)		
	Fos (green)	GluN1 receptor subunit (red)
Primary Ab:	rabbit (SC, sc-52); 1 : 5000	goat (SC, sc-1467); 1 : 700
Secondary Ab:	donkey (I, A21206; AF488); 1 : 1000	donkey (I, A11058; AF594); 1 : 1500
GluN2A (NR2a, NMDA*ε*1) and Fos (N = 6)		
	Fos (green)	GluN2A receptor subunit (red)
Primary Ab:	goat (SC, sc-52G); 1 : 2000	rabbit (SC, sc-9056); 1 : 200
Secondary Ab:	donkey (I, A11055; AF488); 1 : 1000	donkey (I, A21207; AF594); 1 : 1000
GluN2B (NR2b, NMDA*ε*2) and Fos (N = 6)		
	Fos (green)	GluN2B receptor subunit (red)
Primary Ab:	goat (SC, sc-52G); 1 : 2000	rabbit (SC, sc-9057); 1 : 500
Secondary Ab:	donkey (I, A11055; AF488); 1 : 1000	donkey (I, A21207; AF594); 1 : 1000
GluN2C (NR2c, NMDA*ε*3) and Fos (N = 3)		
	Fos (green)	GluN2C receptor subunit (red)
Primary Ab:	goat (SC, sc-52G); 1 : 1000	rabbit (SC, sc-50437); 1 : 200
Secondary Ab:	donkey (I, A11055; AF488); 1 : 1000	donkey (I, A21207; AF594); 1 : 750
GluN2D (NR2d, NMDA*ε*4) and Fos (N = 3)		
	Fos (green)	GluN2D receptor subunit (red)
Primary Ab:	goat (SC, sc-52G); 1 : 500	rabbit (SC, sc-10727); 1 : 100
Secondary Ab:	donkey (I, A11055; AF488); 1 : 1000	donkey (I, A21207; AF594); 1 : 1000
GluN3B (NR3B) and Fos (N = 6)		
	Fos (green)	GluN3B receptor subunit (red)
Primary Ab:	goat (SC, sc-52G); 1 : 1000	rabbit (SC, sc-50474); 1 : 150
Secondary Ab:	donkey (I, A11055; AF488); 1 : 1000	donkey (I, A21207; AF594); 1 : 1000
Substance P		
NK1 and Fos (N = 5)		
	Fos (green)	NK1 receptor (red)
Primary Ab:	rabbit (SC, sc-52); 1 : 1000	guinea pig (M, AB15810); 1 : 2,000
Secondary Ab:	donkey (MP, A21206; AF488); 1 : 500	goat (MP, A11076; AF594); 1 : 500

*Note*. Glutamate receptor subunit names are consistent with IUBCP nomenclature, with common receptor subunit names in parentheses [14].

**Table 2:** Percent colocalization of neurotransmitter receptor subunits with Fos-positive neurons. Percentages for each neurotransmitter receptor were determined by dividing the number of colocalized Fos-positive neurons by the total number of Fos-positive neurons present within each location.

	Superficial MDH (laminae I and II)	Deep MDH (laminae III–V)	Paratrigeminal
AMPA			
GluA1	95.8 ± 1.0	91.7 ± 3.1	93.0 ± 4.5
GluA2/3	88.2 ± 5.0	25.0 ± 25.0	75.0 ± 9.0
GluA4	3.0 ± 3.0	0.0 ± 0.0	11.1 ± 11.1
Kainate			
GluK1/2/3	3.8 ± 2.6	6.1 ± 6.1	0.0 ± 0.0
GluK4	2.9 ± 1.4	4.2 ± 2.8	12.9 ± 4.8
GluK5	0.1 ± 0.1	0.0 ± 0.0	3.4 ± 1.5
NMDA			
GluN1	89.9 ± 2.6	94.4 ± 2.4	95.5 ± 2.4
GluN2A	41.4 ± 3.5	40.1 ± 3.2	57.3 ± 3.8
GluN2B	7.6 ± 3.0	16.2 ± 7.3	15.3 ± 9.6
GluN2C	0.0 ± 0.0	0.0 ± 0.0	0.0 ± 0.0
GluN2D	0.0 ± 0.0	0.0 ± 0.0	0.0 ± 0.0
GluN3B	5.9 ± 1.7	10.8 ± 5.5	7.9 ± 3.7
Substance P			
NK1	64.0 ± 4.1	40.8 ± 2.9	19.5 ± 3.4

## Abbreviations

AEN: Anterior ethmoidal nerve
AMPA: *α*-Amino-3-hydroxy-5-methyl-4-isoxazole-propionate glutamate receptors
BPa: Arterial blood pressure
BSA: Bovine serum albumin
HR: Heart rate
MDH: Medullary dorsal horn
NK1: Neurokinin substance P receptor
NMDA: N-Methyl-D-aspartate glutamate receptors
PBS: Phosphate buffered saline
SE: Standard error
SDH: Spinal dorsal horn
SP: Substance P.
